# Characterisation of an Ovine Keratin Associated Protein (KAP) Gene, Which Would Produce a Protein Rich in Glycine and Tyrosine, but Lacking in Cysteine

**DOI:** 10.3390/genes10110848

**Published:** 2019-10-26

**Authors:** Hua Gong, Huitong Zhou, Jiqing Wang, Shaobin Li, Yuzhu Luo, Jonathan G. H. Hickford

**Affiliations:** 1 International Wool Research Institute, Gansu Agricultural University, Lanzhou 730070, China; Hua.gong@lincoln.ac.nz (H.G.); Huitong.Zhou@lincoln.ac.nz (H.Z.); wangjq@gsau.edu.cn (J.W.); Lisb@gsau.edu.cn (S.L.); 2 Gene Marker Laboratory, Faculty of Agriculture and Life Sciences, Lincoln University, Lincoln 7647, New Zealand; 3 Gansu Key Laboratory of Herbivorous Animal Biotechnology, Faculty of Animal Science and Technology, Gansu Agricultural University, Lanzhou 730070, China

**Keywords:** Keratin-associated protein KAP36-1 gene (*KRTAP36-1*), variation, wool traits, prickle factor, sheep

## Abstract

The keratin-associated proteins (KAPs) are structural components of hair/wool fibres. All of the KAPs identified to date contain cysteine, which is thought to form disulphide bonds cross-linking the keratin intermediate filaments. Here, we report the identification of a KAP gene in sheep that would produce a protein that contains a high proportion (63.2 mol%) of glycine and tyrosine, but would not contain any cysteine. This suggests that other forms of intra- and inter-strand interaction may occur with this KAP, such as interactions via ring-stacking and hydrogen-bonding. The gene was dissimilar to any previously reported KAP gene, and was therefore assigned to a new family, and named *KRTAP36-1*. The *KRTAP36-1* genome sequence was almost identical to some EST sequences from sheep and goat skin follicles, suggesting that it is present and expressed in sheep and goats. A BLAST search of the human genome assembly sequence did not reveal any human homologue. Three variant sequences (named *A* to *C*) of ovine *KRTAP36-1* were identified and four single nucleotide polymorphisms (SNPs) were detected. One SNP was located 32 bp upstream of the coding region, and all of the others were in the coding region and were nonsynonymous. After correcting for potential linkage to the proximal *KRTAP20-1*, variant *B* of *KRTAP36-1* was found to be associated with increased prickle factor (PF) in wool, suggesting that variation in the gene may have the potential to be used as gene marker for breeding sheep with lower PF.

## 1. Introduction

Wool fibre is primarily composed of hard α-keratins. These are cysteine-rich, particularly in their head and tail domains [1]. These α-keratins are assembled into keratin intermediate filaments (KIFs), and then embedded in an inter-filamentous matrix comprised of small proteins called the keratin-associated proteins (KAPs). Three broad groups of KAPs have been defined: the high sulphur (HS) KAPs with less than 30 mol% of cysteine, the ultrahigh sulphur (UHS) KAPs with more than 30 mol% of cysteine and the high glycine-tyrosine (HGT) KAPs with 35–60 mol% glycine and tyrosine [2]. 

The KAP proteins are thought to cross-link KIFs via disulphide bonds [3], but the precise mechanism of linking is still poorly understood. The argument for disulphide cross-linking is in agreement with the observation that the majority (approximately 97.5%) of cysteines in wool are found to be part of disulphide bridges [4] and that most hard α-keratins and KAPs are cysteine rich, even the HGT-KAPs. Despite being more typically rich in glycine and tyrosine, all of the HGT-KAPs identified to date contain cysteine, ranging from 3.2 mol% in ovine KAP8-2 [5] to 14.9 mol% in ovine KAP20-2 [6]. Little is known about whether the HGT-KAPs contribute to cross-linking via cysteine-based disulphide bonding, as the HS- and UHS- KAPs appear to.

There are 17 known KAP gene families (designated as *KRTAPs*), including seven families (KAP6-KAP8 and KAP19-KAP22) that encode the HGT-KAP proteins in humans [7]. Many of the human *KRTAP* orthologs remain unidentified in sheep, with only eight having been characterised to date [2,6,8,9]. Three additional HGT-*KRTAPs* (*KRTAP6-4*, *KRTAP6-5* and *KRTAP8-2*), which are absent in humans, have been identified in sheep [5,10]. This suggests that sheep have more HGT-*KRTAPs* than humans, and support the idea that the HGT-*KRTAPs* play an important role in determining some of the characteristics of the wool fibre.

In sheep, the HGT-*KRTAPs* are clustered on chromosome 1, in a region that is approximately 723-kb in size, and that is between two HS-*KRTAPs* (*KRTAP11-1* and *KRTAP15-1*) [8]. Bioinformatics analysis of this region led us to identify an open reading frame (ORF) that would encode a glycine and tyrosine-rich protein. This is located near to HGT-*KRTAP20-1*, and, in this study, the identity of this ORF was investigated, sequence variation in this ORF was described and its effect on some wool traits is reported.

## 2. Materials and Methods

This research was undertaken in accordance with the Animal Welfare Act 1999 (New Zealand Government) and the collection of sheep blood drops by the nicking of their ears was covered by Section 7.5 Animal Identification, in: Code of Welfare: Sheep and Beef Cattle (2016); a code of welfare issued under the Animal Welfare Act 1999 (New Zealand Government). 

### 2.1. Sheep Investigated and Wool Samples 

A total of 415 sheep were investigated. These included 46 New Zealand (NZ) Romney sheep (sourced from five farms that are not believed to be connected genetically), 48 Merino sheep (sourced from five farms that are not believed to be connected genetically) and 321 Southdown × Merino-cross lambs (sourced from the same farm, but from six sire-lines).

The association studies were carried out on the 321 Southdown × Merino-cross lambs. All these lambs were ear-tagged with an identification number at birth, and their birth dates, birth weights, birth ranks (i.e., whether they were a single, twin or triplet), gender and dam identity were recorded. All the lambs were managed as a single mob on the same farm up to weaning, when they were separated to two mobs based on their gender. They were shorn at twelve months of age. At shearing, the greasy fleece weight (GFW) was measured for each lamb, and a wool sample was collected from the mid-side region for wool trait measurement using International Wool Textile Organisation (IWTO) standardised methods, at the New Zealand Wool Testing Authority Ltd. (NZWTA, Napier, NZ). This included measurement of wool yield (Yield), mean staple length (MSL), mean staple strength (MSS), mean fibre diameter (MFD), fibre diameter standard deviation (FDSD), coefficient of variation of fibre diameter (CVFD), mean fibre curvature (MFC) and prickle factor (PF; the percentage of fibres of diameter greater than 30 microns). Lamb clean fleece weights (CFWs) were calculated from the GFW and Yield measurements.

A sample of blood from each sheep was collected onto TFN paper (Munktell Filter AB, Sweden) and genomic DNA was purified using a two-step washing techniques detailed in Zhou et al. [11].

### 2.2. PCR Amplification of the Newly Identified Open Reading Frame

A 174-bp ORF that appeared to encode a glycine and tyrosine-rich protein was identified near ovine *KRTAP20-1*, at position nt123318135–123318308 (NC_019458.2) on chromosome 1. Sequences flanking this ORF were used to design two PCR primers to amplify a 367-bp fragment spanning the entire ORF. These primers were 5’-GGTTTACCACACCCACAATG-3’ and 5’-GTAGCATAGCAAGAGTGAAG-3’, and they were synthesised by Integrated DNA Technologies (Coralville, IA, USA).

PCR amplification was performed in a 15-μL reaction containing the genomic DNA on one 1.2-mm punch of TFN paper, 0.25 μM of each primer, 150 μM of each dNTP (Eppendorf, Hamburg, Germany), 2.5 mM of Mg^2+^, 0.5 U of Taq DNA polymerase (Qiagen, Hilden, Germany) and 1× the reaction buffer supplied with the enzyme. The thermal profile consisted of an initial denaturation for 2 min at 94 °C, followed by 35 cycles of 30 s at 94 °C, 30 s at 60 °C and 30 s at 72 °C, and with a final extension of 5 min at 72 °C. Amplification was carried out in S1000 thermal cyclers (Bio-Rad, Hercules, CA, USA).

### 2.3. Screening for Sequence Variation and Variant Sequencing

PCR amplicons were subject to SSCP analysis to screen for sequence variation. A 0.7 μL aliquot of each amplicon was mixed with 7 μL of loading dye (98% formamide, 10 mM EDTA, 0.025% bromophenol blue, 0.025% xylene-cyanol). After denaturation at 95 °C for 5 min, samples were placed rapidly on wet ice and then loaded on 16 cm × 18 cm, 14% acrylamide: bisacrylamide (37.5:1) (Bio-Rad) gels. Electrophoresis was performed using Protean II xi cells (Bio-Rad), at 300 V for 18 h at 11 °C in 0.5 × TBE buffer. The gels were silver-stained by the method described by Byun et al. [12].

PCR amplicons representative of different SSCP patterns from sheep that appeared to be homozygous were sequenced at the Lincoln University DNA Sequencing Facility. For those variants that were only found in heterozygous sheep, they were sequenced using a rapid approach described previously [13]. In this approach, a band corresponding to the variant was excised as a gel slice from the polyacrylamide gel, macerated and then used as a template for reamplification with the original primers. This second amplicon was then sequenced. 

### 2.4. Sequence Analyses

Sequence alignments, translations, comparisons and the construction of phylogenetic tree were carried out using DNAMAN (version 5.2.10, Lynnon BioSoft, Vaudreuil, Canada). The BLAST algorithm was used to search the NCBI GenBank (www.ncbi.nlm.nih.gov/) databases for homologous sequences. 

### 2.5. Genotyping of KRTAP20-1

The 321 Southdown × Merino-cross lambs used for the association analyses were also genotyped for variation in *KRTAP20-1* using a PCR-SSCP technique described previously [8]. Briefly, *KRTAP20-1* was amplified using the PCR primers 5’-TCATATTCTGCAAGCAAAGGC-3’and 5’-GCTGATGGGTCTCAGTCAC-3’. After denaturation, amplicons were electrophoresed using 14% acrylamide: bisacrylamide (37.5:1) (Bio-Rad) gels containing 1.0% *v/v* glycerol, at 8 °C and 390 V for 18 h. Polymerase chain reaction amplicons of the previously described variants [8] were included as references to determine genotypes in the gels.

### 2.6. Statistical Analyses of Associations 

All the statistical analyses were undertaken using Minitab version 16 (Minitab Incorporated, State College, PA, USA).

General linear mixed-effect models (GLMMs) were employed to individually evaluate the effect of the presence or absence (coded as “1” or “0”) of the three variants of the ORF (*A*, *B* and *C*), on the ten wool traits that had been measured or calculated. In these models, gender and sire were included as fixed and random factors respectively, as they affected all of the wool traits. Differences in the marginal means derived from these models were considered to be significant when *p* < 0.05, and trends were noted when 0.05 ≤ *p* < 0.10.

As a consequence of variants occurring in genotypes, it is possible that the effect of one variant in the genotype is affected by the presence of the other variant in that genotype. Accordingly, any variant sequence of the ORF in the initial GLMMs, which had an association with a wool trait of *p* < 0.200 and thus was potentially associated with the trait (albeit at a low threshold), was included as an explanatory factor in a second set of multivariant presence/absence models. Once again, gender and sire were included as fixed and random factors respectively in this second set of models, as they affected all the wool traits. Differences in the marginal means derived from these models were once again considered to be significant when *p* < 0.05, and trends were noted when 0.05 ≤ *p* < 0.10.

Finally, given that variation in the nearby gene *KRTAP20-1* has been described as affecting wool yield and mean fibre diameter-associated traits [8], and to test whether the associations identified above between the ORF variants and variation in the wool traits was as a consequence of proximity to *KRTAP20-1*, a third set of GLMMs that included *KRTAP20-1* genotype as an explanatory factor, were subsequently undertaken. Once again, gender and sire were included as fixed and random factors respectively in this third set of models, as they affected all the wool traits. Differences in the marginal means derived from these models were once again considered to be significant when *p* < 0.05, and trends were noted when 0.05 ≤ *p* < 0.10.

Birth rank was not found to affect the ten wool traits and thus it was not included as an explanatory factor in any of the above models. 

## 3. Results

### 3.1. Identification of KRTAP36-1 in Sheep and the Absence of a Homologue in the Human Genome

**Figure 1:**
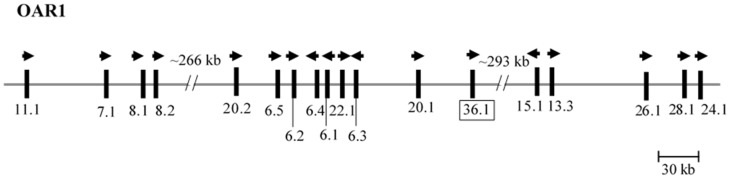
*KRTAPs* identified on the sheep chromosome 1 region that contains a newly identified *KRTAP36-1*. The newly identified gene is shown in a box, and the 17 previously identified *KRTAPs* are also shown. Vertical bars represent the location of different *KRTAPs* and the arrowheads indicate the direction of transcription. The numbers below the bars indicate the name of the respective KAP genes (i.e., 11.1 is *KRTAP11-1*). The nucleotide distances are approximately and refer to NC_019458.2.

The ORF at nt123318135–123318308 (NC_019458.2) was located between *KRTAP20-1* and *KRTAP15-1* on sheep chromosome 1 (Figure 1). This ORF had a nucleotide sequence that was different to all of the ovine *KRTAPs* identified to date, but shared 99% identity to two GenBank sequences labelled as ovine *KRTAP16-1* (KF543056.1) and caprine *KRTAP16-1* (AY502950.1). A BLAST search of the NCBI Expressed Sequence Tag (EST) database, revealed that this ORF sequence had 99% identity to eight ovine mRNA sequences (JK724590.1, GO705930.1, EE851605.1, EE847453.1, EE753136.1, GO779858.1, EE848868.1 and EE848117.1), and 98% identity to one caprine mRNA sequence (CD052106.1) derived from skin tissue/wool follicle. This suggests that this ORF is expressed in the wool follicle and the sequence differences between this ORF and ovine ESTs may reflect sequence variation, or errors in RT-PCR and/or genomic sequencing.

The ORF was predicted to encode a protein of 57 amino acid residues. Five amino acids were common (totalling 93 mol%) in this protein, with the most common being glycine (35.1 mol%), followed by tyrosine (28.1 mol%), serine (14.0 mol%), leucine (8.8 mol%) and phenylalanine (7.0 mol%). The protein would not contain any cysteine, which excludes it from being assigned to either the HS- or UHS-KAP groups. 

**Figure 2:**
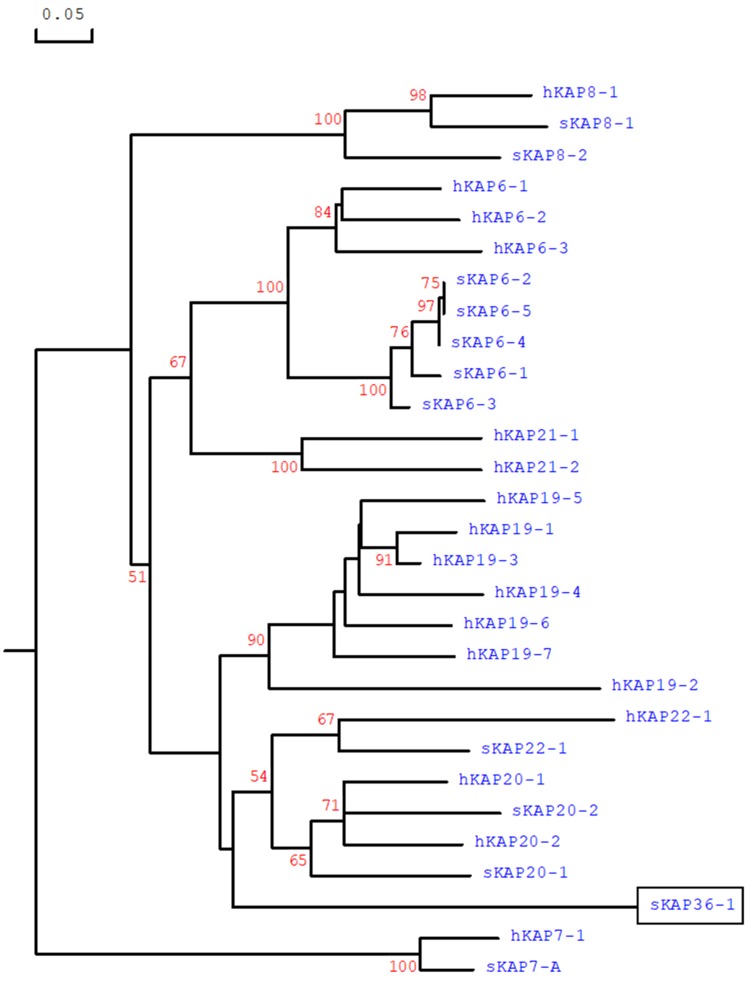
Phylogenetic tree of the HGT-KAPs identified in sheep and human. The tree was constructed using the predicted amino acid sequences. The numbers at the forks indicate the bootstrap confidence values and only those equal to or higher than 50% are shown. The sheep KAPs are indicated with a prefix “s”, whereas the human sequences are indicated with “h”. The newly identified ORF is designated as sheep KAP36-1 gene and is indicated in box. The GenBank accession numbers for other sheep HGT-KAPs are NM_001193399 (sKAP6-1), KT725832 (sKAP6-2), KT725837 (sKAP6-3), KT725840 (sKAP6-4), KT725845 (sKAP6-5), X05638 (sKAP7-1), X05639 (sKAP8-1), KF220646 (sKAP8-2), MH243552 (sKAP20-1), MH071391 (sKAP20-2) and KX377616 (sKAP22-1). The GenBank accession numbers for human HGT-KAPs are: NM_181602 (hKAP6-1), NM_181604 (hKAP6-2), NM_181605 (hKAP6-3), AJ457063 (hKAP7-1), AJ457064 (hKAP8-1), AJ457067 (hKAP19-1), NM_181608 (hKAP19-2), NM_181609 (hKAP19-3), NM_181610 (hKAP19-4), NM_181611 (hKAP19-5), NM_181612 (hKAP19-6), NM_181614 (hKAP19-7), NM_181615 (hKAP20-1), NM_181616 (hKAP20-2), NM_181619 (hKAP21-1), NM_181617 (hKAP21-2) and NM_181620 (hKAP22-1).

Phylogenetic analysis of this ORF and all of the HGT-KAP genes identified to date, revealed that it was separated from all known HGT-KAP families and the distance of separation suggested it should be designated as a new KAP family (Figure 2). Despite only 28 KAP families (KAP1 to KAP28) have been confirmed across mammalian species [2,7,14], the names KAP29-KAP35 have been used for some sequences reported in public databases. In this context and to avoid confusion this ORF was named SHEEP-*KRTAP36-1*, according to the updated *KRTAP*/KAP nomenclature [15].

A BLAST search of the human Genome Assembly GRCh38.p13 using this ORF did not reveal any homologue in the human genome, and the closest similarity (84%) was to *KRTAP19-3*.

### 3.2. Variation in Ovine KRTAP36-1

None of the ovine EST sequences were identical to the ORF sequence reported in the sheep assembly sequence, with each having one or two nucleotides different when compared to the ORF sequence, and with a total of four nucleotide differences being observed at positions c.16, c.23, c.75 and c.-32.

**Figure 3:**
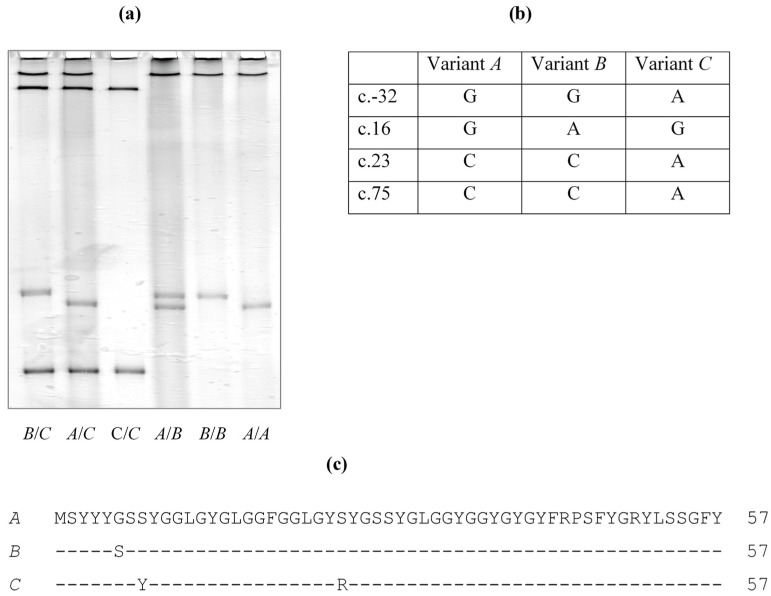
Polymorphism of ovine *KRTAP36-1*. (**a**) Three variants (*A* to *C*) in either homozygous or heterozygous forms were detected by PCR-SSCP. (**b**) Four single nucleotide polymorphisms (SNPs) were found in these variants. (**c**) Three of the SNPs would lead to amino acid changes and result in three different amino acid sequences.

To determine whether these EST nucleotide differences result from sequence variation in the gene, potential variation in ovine *KRTAP36-1* was screened for using a PCR-SSCP approach. Three banding patterns representing three variants (*A* to *C*) were detected (Figure 3) and four single nucleotide polymorphism (SNPs) were detected, including one SNP (c.-32G/A) upstream of the coding region, and three SNPs (c.16G/A, c.23C/A and c.75C/A) in the coding region. All of the coding SNPs were nonsynonymous and would result in the amino acid substitutions p.Gly6Ser, p.Ser8Tyr and p.Ser25Arg. These coding region SNPs match well with three (c.16, c.23 and c.75) of the four nucleotide differences between the ORF sequence and the sheep genome assembly, and with the EST sequences. These variant sequences were deposited into GenBank with accession numbers MK770620-MK770622.

All of the *KRTAP36-1* variants were found in the Merino and Romney breeds, but at different frequencies. Of the 48 Merino sheep, three were *AA*, five were *AB*, ten were *AC*, four were *BB*, 14 were *BC* and 12 were *CC*, with variants *A*, *B* and *C* being present at frequencies of 21.9%, 28.1% and 50.0%, respectively. Of the 46 Romney sheep, one was *AA*, eight were *AB*, three were *AC*, four were *BB*, 15 were *BC* and 15 were *CC*, with frequencies of 14.1%, 33.7% and 52.2% being detected for variants *A*, *B* and *C*, respectively. 

Of the four SNPs identified, three (c.-32G/A, c.23C/A, and c.75C/A) were found to be in linkage. Near to these SNPs, a Chi-like sequence (5’-GCTGGTGA-3’) was found at positions c.-66 to c.-59. 

### 3.3. Effect of KRTAP36-1 Variation on Wool Traits

When only *KRTAP36-1* was considered in GLMMs, the presence of variant *A* was found to be associated with increased GFW. Variant *B* was found to be associated with decreased PF in the single-variant GLMMs, but the association disappeared in the multivariant GLMMs. Variant *C* was associated with an increase in MFD and PF, and the association with PF persisted in the multivariant GLMMs (Table 1). 

Given that variation in *KRTAP20-1* (a gene located near to *KRTAP36-1*) has been reported to affect wool weight and mean fibre diameter-associated traits [8], and to test whether the associations detected above were because of the effect of *KRTAP20-1*, the GLMMs were then corrected for variation in *KRTAP20-1*. All of the associations disappeared or became a trend, except for the association between *KRTAP36-1* and PF, where the association persisted (Table 1). 

## 4. Discussion

This study has identified a new KAP gene on sheep chromosome 1. This gene is comprised of one exon, appears to be expressed, and the protein encoded for is rich in glycine and tyrosine. The gene is clustered with all of the other known HGT-KAP genes on sheep chromosome 1, but it does not share high sequence similarity to any known HGT-KAP gene. It would however appear to be phylogenetically related to the HGT-KAP genes (Figure 2).

These characteristics led us to identify this gene as a HGT-KAP gene, and to assign it into a new KAP family. While two GenBank sequences (KF543056.1 and AY502950.1) that are similar to this sequence are designated as *KRTAP16-1*, the assignment of these sequences into the KAP16 family is inappropriate, as KAP16 is a HS-KAP family, and in sheep the name has already been used for other HS-KAP genes [16] that are not related to this newly identified HGT-KAP gene. This gene was therefore named SHEEP-*KRTAP36-1*, this being a KAP family name that has not been used previously.

The gene is located in a chromosome region near to *KRTAP20-1*, a gene for which the location and transcription direction differ between sheep and humans [8]. This suggests this region of the chromosome may have evolved via different pathways in sheep and humans, and thus it is perhaps not surprising that *KRTAP36-1* is present in sheep and goats, but absent in humans.

The protein (KAP36-1) putatively produce by this gene appears to possess a high content (63.2 mol%) of glycine and tyrosine, but it does not contain cysteine. Cysteines are commonly found in keratins and KAPs, and they are thought to form disulphide bonds that cross-link the KIFs and KAPs. The absence of cysteine in KAP36-1, suggests that the other forms of cross-linking may occur. In this respect, tyrosine is an aromatic amino acid containing a benzene ring. The possession of this stable ring structure may allow tyrosine to interact with other tyrosine residues and other aromatic amino acids via a ring-stacking mechanism. This has been reported for other aromatic amino acid-containing proteins [17]. In the HGT-KAPs, the tyrosine residues are usually surrounded by glycine residues. Having the smallest residue (glycine) in proximity to the tyrosine, will allow the tyrosine residues greater conformational freedom to move their benzene rings into a preferred orientation, and thus enable the formation of stronger amino acid to amino acid interactions. Tyrosine also possesses a hydroxyl group, which can act as a hydrogen donor and form hydrogen bond interactions with the centre of the benzene ring from another tyrosine, or other aromatic amino acids [18]. This would make the ring-stacking interaction even stronger. This kind of interaction is expected to result in the wool fibre being strengthened, while simultaneously giving some degree of pliability [19].

Unlike the covalent disulphide bonding, ring-stacking and hydrogen bonding do not require any additional covalent bond formation and they could readily form soon after the proteins are synthesised. Therefore, we hypothesise that these types of interaction may serve as the primary interactions that cross-link the KIFs and stabilise the wool fibre structure, and that this occurs prior to the formation of disulphide bonds. This is in agreement with the observation that the HGT-KAPs are expressed first among the KAPs in the wool follicle [20], and immediately following intermediate filament synthesis. In that capacity, they may therefore play a key role in the assembly of KIFs, and hence act as a key determinant of fibre structure.

When the effect from a nearby *KRTAP* was corrected for, there was a loss or weakening of the associations with GFW, MFD and FDSD. This suggests that these associations are not due to the effect of *KRTAP36-1*, but instead result from the linkage with other *KRTAPs* nearby. This highlights the importance of correcting for the effect of other *KRTAPs* that are in proximity on the same chromosome, and such that a more precise indication of how any given gene may be affecting wool traits can be obtained. The persistence of the association with PF after the correction for the effect of *KRTAP20-1* variation (which has also been shown to affect PF), strengthens the finding.

Though the potential functional effect of the SNP in the 5’-UTR should not be ignored, as 5’-UTR SNPs may affect gene expression [21], it is interesting to note that all of the coding region SNPs were nonsynonymous. Of these, two were in linkage with the 5’-UTR SNP. The only coding-region SNP that was not in the linkage with other SNPs was c.16G/A, which was the only sequence difference between variants *A* and *B*.

Variant *B* was found to be associated with a high PF, but variant *A* was not. The SNP c.16G/A would result in the substitution of glycine by serine at the 6^th^ amino acid residue in the protein encoded by variant *B*, with a string of three tyrosine residues at positions 3 to 5. The substitution of glycine by serine at position 6 may have an impact on the conformational freedom of this string of tyrosine residues, with this potentially impacting ring-stacking and/or hydrogen bonding, and with a consequent reduction in fibre compactness or density, and consequently a higher PF.

The percentage of fibres over a given diameter threshold (typically 30 microns), is an indicator of the relative comfort of wool fibres worn next to the skin. Fibres over 30 microns in diameter tend to bend less and produce a “prickle” sensation on the skin’s surface, and with more than 5% of the total number of fibres, the effect tends to be quite noticeable (SGS Wool Testing Services 2011) [22]. The finding of association between *KRTAP36-1* and PF suggest that *KRTAP36-1* has potential to be used as a gene marker for breeding sheep to produce wool with reduced PF, and that this could add value to fine wool production. 

Sheep have primary and secondary wool follicles. The fibres produced by secondary follicles are finer while the fibres produced by primary follicles are usually much larger. The observation that *KRTAP36-1* variation is only associated with variation in PF, and no other fibre diameter-associated traits such as MFD, FDSD and CVFD, suggests that *KRTAP36-1* may affect or reflect the secondary to primary wool follicle ratio (S/P ratio), and that this consequently affects PF. This would require confirmation with an analysis of the S/P ratios in sheep carrying the different variants of *KRTAP36-1*. A putative function for *KRTAP36-1* in regulating or determining S/P ratios, or as a consequence of variation in S/P ratio that has come about for another reason, may explain the absence of this gene in humans, as humans only have one type of follicle.

Merino sheep usually have a higher S/P ratio and the wool produced has a lower PF than Romney sheep. In the sheep populations investigated in this study, *B* was found at a higher frequency in Romney sheep, than Merino sheep. This supports the contention that variant *B* of *KRTAP36-1* was associated with increased PF.

The presence of four SNPs in a 327-bp PCR fragment (excluding the primer binding regions) corresponds to a density of 12.2 SNPs per kb. This is much higher than the average density of 4.9 SNPs per kb across the sheep genome suggested by Kijas et al. [23]. This suggests that *KRTAP36-1* has far less functional constraint than other parts of the genome. This is consistent with the trend reported for many other *KRTAP*s [24,25,26,27,28]. Little is known of how the variation in *KRTAP*s has come about, but the lineage of SNPs and the presence of a Chi-like sequence suggests that gene conversion or nonreciprocal genetic exchange may have occurred in *KRTAP36-1*. Gene conversion or nonreciprocal genetic exchange has been suggested previously to be a mechanism for generating variation in other *KRTAPs* [24,29,30].

The HGT-*KRTAPs* are clustered on one chromosome region surrounding by HS*-KRTAPs*. With the identification of *KRTAP36-1*, the number of HGT-*KRTAPs* identified in sheep has risen from eleven to twelve. Of these, the effect on wool traits has been investigated for eight HGT-*KRTAPs*: *KRTAP6-1* [31,32], *KRTAP6-3* [33], *KRTAP8-1* [34], *KRTAP8-2* [35], *KRTAP20-2* [6], *KRTAP20-1* [8], *KRTAP22-1* [9] and now *KRTAP36-1*. In Merino-cross sheep, *KRTAP6-1* is reported to affect wool yield, MFD, FDSD, CVFD and PF [31]; *KRTAP6-3* is reported to affect MFD, FDSD and PF [32]; *KRTAP8-1* is reported to affect wool fibre staple strength and curvature [34]; *KRTAP22-1* is reported to affect wool yield [9], *KRTAP20-2* is reported to affect MFC [6], *KRTAP20-1* is reported to affect GFW, wool yield, MFD, FDSD and PF [8]; and, in this study, *KRTAP36-1* is found to affect PF. In the early life of Chinese Tan sheep, *KRTAP6-1* is reported to affect wool growth, crimp number and the degree of crimping [32], while *KRTAP8-2* is found to affect wool growth and degree of crimping [35].

The finding that the HGT-*KRTAPs* have similar but unique effects on wool traits, suggests that all the HGT-*KRTAPs* may contribute to cross-linking roles in the wool fibre, but with different HGT-*KRTAPs* contributing differently to the extent, timing and/or mechanisms of cross-linking. 

## Figures and Tables

**Table 1:** Association between the absence or presence of *KRTAP36-1* variants and various wool traits.

Trait ^1^	Variant Assessed ^2^	Other Variants Fitted	Other *KRTAP* Fitted	(Mean ± SE) ^3^		*P*
				Absent	Present	
GFW	*A*	None	None	2.39 ± 0.04	2.49 ± 0.04	0.035
(kg)	*B*	None	None	2.46 ± 0.04	2.43 ± 0.04	0.616
	*C*	None	None	2.46 ± 0.04	2.44 ± 0.04	0.680
	*A*	None	*KRTAP20-1*	2.44 ± 0.05	2.49 ± 0.04	0.345
CFW (kg)	*A*	None	None	1.76 ± 0.03	1.81 ± 0.03	0.172
	*B*	None	None	1.79 ± 0.03	1.79 ± 0.03	0.968
	*C*	None	None	1.81 ± 0.04	1.78 ± 0.03	0.359
Yield	*A*	None	None	73.2 ± 0.62	72.2 ± 0.56	0.133
(%)	*B*	None	None	72.4 ± 0.56	72.8 ± 0.62	0.572
	*C*	None	None	72.7 ± 0.65	72.5 ± 0.54	0.742
MSL (mM)	*A*	None	None	83.2 ± 1.22	83.6 ± 1.11	0.723
	*B*	None	None	83.3 ± 1.11	83.7 ± 1.23	0.765
	*C*	None	None	83.0 ± 1.28	83.7 ± 1.08	0.634
MFD	*A*	None	None	19.5 ± 0.18	19.5 ± 0.16	0.913
(µM)	*B*	None	None	19.6 ± 0.16	19.4 ± 0.18	0.208
	*C*	None	None	19.3 ± 0.19	19.7 ± 0.16	0.025
	*C*	None	*KRTAP20-1*	19.7 ± 0.20	20.0 ± 0.17	0.081
FDSD	*A*	None	None	4.10 ± 0.06	4.10 ± 0.06	0.922
(µM)	*B*	None	None	4.13 ± 0.06	4.04 ± 0.06	0.164
	*C*	None	None	4.01 ± 0.07	4.14 ± 0.06	0.053
	*C*	*B*	None	4.01 ± 0.07	4.13 ± 0.06	0.099
	*C*	*B*	*KRTAP20-1*	4.12 ± 0.07	4.22 ± 0.06	0.179
CVFD	*A*	None	None	20.9 ± 0.23	20.9 ± 0.21	0.930
(%)	*B*	None	None	21.0 ± 0.21	20.8 ± 0.23	0.403
	*C*	None	None	20.8 ± 0.24	21.0 ± 0.20	0.395
MSS	*A*	None	None	23.7± 0.79	23.8 ± 0.72	0.951
(N/ktex)	*B*	None	None	24.2 ± 0.72	23.1 ± 0.80	0.190
	*C*	None	None	23.7 ± 0.83	23.8 ± 0.70	0.876
PF	*A*	None	None	2.72 ± 0.32	2.57 ± 0.29	0.681
(%)	*B*	None	None	2.91 ± 0.29	2.25 ± 0.32	0.061
	*C*	None	None	2.04 ± 0.33	2.99 ± 0.28	0.007
	*B*	*C*	None	2.69 ± 0.31	2.31 ± 0.32	0.765
	*C*	*B*	None	2.10 ± 0.33	2.91 ± 0.29	0.031
	*C*	*B*	*KRTAP20-1*	2.71 ± 0.37	3.49 ± 0.32	0.038
CURV	*A*	None	None	87.9 ± 1.61	88.3 ± 1.47	0.817
(°/mM)	*B*	None	None	88.3 ± 1.46	88.0 ± 1.62	0.891
	*C*	None	None	86.9 ± 1.68	88.9 ± 1.42	0.269

^1^ GFW: Greasy fleece weight; CFW: Clean fleece weight; Yield: wool yield; MFD: Mean fibre diameter; FDSD: Fibre diameter standard deviation; CVFD: Coefficient of variation of fibre diameter; MSL: Mean staple length; MSS: Mean staple strength; CURV: Curvature; PF: Prickle factor (percentage of fibres over 30 microns). ^2^ Of the 321 Southdown × Merino-cross lambs, *KRTAP36-1* variant *A* was present in 195 lambs and absent in 126 lambs, variant *B* was present in 139 lambs and absent in 182 lambs, and variant *C* was present in 194 lambs and absent in 127 lambs. ^3^ Predicted means and standard error of these means derived from GLMMs with various factors being included into the models for different wool traits as described in Materials and Methods.
